# Microbiological analysis and bioremediation bioassay for characterization of industrial effluent

**DOI:** 10.1038/s41598-022-23480-7

**Published:** 2022-11-07

**Authors:** Nidal Mohammed Omar Zabermawi, Amani H. Alyhaiby, Ebtesam A. El-Bestawy

**Affiliations:** 1 Department of Biological Sciences, Microbiology, Faculty of Sciences, King Abdulaziz University, Jeddah, 21589 Saudi Arabia; 2 Department of Environmental Studies, Institute of Graduate Studies and Research, Alexandria University, 163 Horria Ave. El-Shatby, P.O. Box 832, Alexandria, Egypt

**Keywords:** Biological techniques, Microbiology

## Abstract

This study aims to investigate bacteria for biodegradation of oil pollutants from oily industrial wastewater to be used as bioremediation tools and to determine the characterization of bioremediation bioassays. A screening bioassay was carried out using six exogenous environmental bacterial strains to degrade oily pollution, which indicated promising clearance of the oily wastewater. Two strains, namely Enterobacter cloacae 279-56 (R4) and Pseudomonas otitis MCC10330 (R19), could successfully eliminate oil content and reasonable removal of the organic load. Results showed that the two promising bacterial candidates (R4 and R19) were selected according to the preliminary screening of the six tested bacteria considered the most efficient for all the tested parameters. The highest Removal Efficiency (Removal Efficiency resulted in Residual levels of total dissolved solids (TDS), biochemical oxygen demand, chemical oxygen demand, and Oil content in the treated oily wastewater effluents are 1940, 171, 131, and 84 mg/l respectively where these results are not within safe discharge limits, except for TDS. Hence, the bioremediation assays were carried out using the mixed culture since it was the most efficient strain for degrading all tested parameters.

## Introduction

The expansion of a nation's economy is mostly attributable to its industrial sector. On the other hand, an imminent threat has emerged due to the proliferation of companies that pollute the environment with their effluent. Toxic industrial waste is a major contributor to drinking water shortages, with negative consequences for human health, agricultural productivity, ecosystem health, economic vitality, and social and aesthetic quality^1^. Large amounts of crude oil products, polycyclic and aromatic hydrocarbons, metal derivatives, sulfides, surface-active compounds, naphthalenic acids, phenols, and other chemicals are present in waste water from the petrochemical and crude oil processing industries. Due to inefficient purification methods, waste water may become very hazardous, accumulating toxic waste in receiving water bodies with detrimental effects on the ecology. The health of aquatic organisms and the contaminants in refinery effluent and wastewater are positively correlated, according to several research. Prior research suggests a connection between fish health issues, sediment containing aromatic hydrocarbons from refinery wastes, and water pollution^2^.

Oil spills usually happen through accidents during transportation, pumping, and refining. Since oil hydrocarbons (aromatic and aliphatic hydrocarbons) pose an adverse impact on human health and the environment, they are considered priority environmental pollutants by the US Environmental Protection Agency. Biological treatment has been considered a cost-effective, efficient, and environmentally sound method for the degradation of hydrocarbons in petroleum contaminants^3^. Many technologies, including chemical, physical, and biological approaches, have been developed for contaminated site rehabilitation. Physical and chemical approaches are expensive and may also bring additional waste, and in many cases, they just turn pollutants from one stage to another. Using living organisms to rehabilitate contaminated sites, biological treatment has attracted great interest in research over the past decade as a cost-effective and viable alternative to chemical approaches. Biodegradation by natural bacterial and fungal populations is well recognized as many microorganisms can use hydrocarbons for energy and carbon sources. These microorganisms completely decompose pollutants through several intracellular and extracellular enzymatic activities, with CO2 as the final precursor. The mineralization rate and extent depend on the microorganisms' metabolic capacity^4^. A secondary wastewater treatment method known as biological treatment makes use of the metabolic processes of microorganisms to oxidize or decrease inorganic and organic components and convert them into dense biomass that can then be removed via the sedimentation process^5^. Besides being cost-effective, it results in the degradation or complete mineralization of pollutants. It may refer to the complete degradation of organic pollutants to carbon dioxide, water, and inorganic compounds. The principle of the biological treatment process is to take advantage of petroleum hydrocarbons as the sole origin of carbon and energy. Naturally occurring hydrocarbon-degrading bacteria either consume or absorb all harmful components of the pollutants, cleaning the surrounding area and producing a pollution-free region. Sometimes native bacterial population is not sufficient to remove contaminants quickly. In these cases, the rate of biodegradation can be sped up by adding a special mix of strains or strains grown in a lab that can break down these contaminants quickly. This is called "bio-augmentation." Oil bioremediation benefits from the high efficiency of treatment, relatively quick action, low cost, and applicability in both in situ and ex-situ. On the other hand, the introduction of hydrocarbon-degrading bacteria in sites contaminated by oil does not guarantee to remove of all of the crude oil components because some of the components are still difficult to decompose, such as polycyclic aromatic hydrocarbons (PAHs) and alkanes of shorter and longer chains (< C10 and C20–C40)^6^. For effective wastewater treatment, Hesnawi et al.^7^ emphasized the requirement for highly specialized strains.

Effective and ecologically safe organic waste treatment technologies are required to fight the extra environmental loads^8,9^. To attain high treatment efficiency, bioengineered methods customized for each kind of hazardous and organic waste are needed. When assessing the effectiveness of a wastewater treatment system, it is crucial to first assess and characterize the wastewater in question. Drug manufacturers now use a wide range of therapeutic approaches, from the purely chemical and physicochemical to the purely biological and even the purely sophisticated oxidative^10,11^. Bioremediation is a cutting-edge and extensively used method for cleaning up effluent from the pharmaceutical industry. While there has been some research on characterization-assisted bioremediation techniques, it is limited to the context of one locale. Thus, the current study recognizes the necessity to investigate the region in light of the gaps and dearth of prior studies. The present study aimed to investigate the bioremediation bioassay to characterize industrial effluent using different bacterial strains.

## Material and Methods

An experimental study was conducted from 2018 to 2019 at King Abdulaziz University,Jeddah.Saudi Arabia. Samples of wastewater were collected during the study course from the final drainage of oil refinery polluted effluents located in Alexandria, Egypt. Six species of exogenous bacterial members were selected based on their excellent biodegradation capabilities towards organic contaminants. They include Pseudomonas stutzeri (PS), Pseudomonas aeruginosa PAO1 (R3), Enterobacter cloacae 279-56 (R4), Pseudomonas balearica SP1402 (R7), Pseudomonas otitidis MCC10330 (R19) and Bacillus cereus ATCC 1579 (R23). The selected bacterial species were investigated as individual and mixture, free and fixed cultures to remediate oil-contaminated effluents. Among them, five species (R3, R4, R7, R19 and R23) were provided from previous bioremediation study in the Faculty of Science, King Abdul Aziz University where they exhibited high bioremediation capabilities for heavily contaminated soil^12^, while Pseudomonas stutzeri (PS) was provided from the Institute of Graduate Studies & Research, Alexandria University (IGSR) collection^13^.

### Media Preparation

Dehydrated nutrient broth (NB) and agar (NA) were supplied by HIMEDIA and used during the present study. NB medium contained (g/l) Peptic digest of animal tissue, 5 g Sodium chloride, 5 g Yeast extract, 1.5 g and Beef extract, 1.5 g. NA medium contained the same ingredients as NB with the addition of 15.0 g/l Agar. They were prepared by dissolving 13 and 28 g/l from NB and NA dehydrated media respectively, pH was adjusted to 7.4, sterilized by autoclaving at 121ºC for 20 min and freshly used for growth experiments as well as biodegradation assays.

### Culturing of Bacteria

Exogenous bacteria were cultured, including inoculation in liquid and solid media. Then, organisms that grew on solid culture were removed, purified by streaking culturing, and stocks of the pure cultures were prepared and incubated for 24 h at 37 °C.

### Determination of the Total Viable Count of Bacteria (TVCB)

For the determination of TVC, pour plate technique of the standard plate count method was used^14^. In order to get a reasonable count per plate (30 to 300 colony/plate), samples were subjected to serial dilutions. Specific volume (100, 500, or 1000 µL) from the final dilution, was cultured under aseptic situations into petri dish (3 replica each) to which 20 ml nutrient agar (NA- enrichment medium) poured in order to allow the growth of aerobic and facultative microbes. After that, plates were left for 15 min to dry, then inverted and incubated for 24 h at 37ºC. CFU of the TVC was recorded and averages were calculated.

#### Isolation and Purification

Purifying heterotrophic bacterial colonies was performed in single or repeated steps of culturing and re-culturing by streaking on NA agar plates and then incubating at 37 °C till obtaining pure isolates were obtained. These pure isolates were inoculated onto NA slants, then incubated and preserved in the fridge as a stock for further investigations.

In order to select the most promising candidates for oil contaminated effluent bioremediation process, the pure exogenous isolates were investigated visually daily for a month. This process was done for each candidate individually in 250 ml flasks containing oil contaminated effluent and incubated at 37 °C under 100 rpm agitation speed. Based on the preliminary screen, the six selected species (R3, R4, R7, R19, R23 and PS) indicated promising clearance of the oily wastewater, therefore, they were considered as potential candidates. They were tested for remediation of the highly oil contaminated effluent as free living individuals or mixed cultures where they inoculated in 100 ml NB medium and incubated till heavy growth was obtained. Then, the total viable count of bacteria (TVC) was counted in order to define the density of the different inocula at the start time. Oily wastewater tested was characterized immediately after sampling collection, in order to define its pollution strength (raw readings) and dispensed into 7 aliquots of 600 ml each in 1 L flasks to which individual and mixed cultures were inoculated. In addition to the bacterial amended wastewater, one flask with the same wastewater volume was left without bacterial augmentation and considered as a control. Then all cultures (bacteria and bacteria-free) were incubated at room temperature (≈ 26 °C) 7 days under agitation. Samples were aseptically drawn at 24-h intervals and the selected parameters were determined for the treated effluents in order to define their residual levels at each exposure time. Then, the removal efficiency was calculated for the treated effluents to define the effectiveness of remediation process.

According to the removal efficiency in the screening bioassay; R4 and R19 showed the most degradation capability of oily wastewater contaminants. They were reactivated overnight on nutrient agar (NA) medium prior to each experiment. Bioremediation enhancement was performed using R4 and R19 as individual and mixed cultures. The bioassay was performed by separate activation of R4, R19 and their mixed culture in 100 ml nutrient broth and incubation until heavy growth was obtained (≈ 24 h). Cultures were then placed in a sterilized 1000 ml conical flask containing 500 mL of oil-contaminated wastewater. In addition, 500 raw oily waste water was placed in another flask without seeding of any bacteria and acts as control. The seeded and un-seeded samples were incubated at room temperature for 7 days where samples were drawn at 24 h interval. Characterization of the wastewater was performed before and after treatment and the efficiency of removal was calculated.

Based on the results of the free living bioassay and the removal efficiency of the investigated parameters, bacterial consortium (*Enterobacter cloacae* 279-56 (4), and *Pseudomonas otitidis* MCC10330 (R19) were selected to be fixed on a gravel aggregates as a biofilm since they showed the broad and highest degradation activity and capability for remediating the contaminants in the oily effluent compared to other tested bacteria. The selected bacterial consortium was maintained on nutrient agar (NA) medium and prior to each experiment, the culture was reactivated in NB overnight. Indigenous bacteria present in the wastewater were examined in the control system.

#### Characterization of the Raw and Treated Industrial Effluent

Oily wastewater was characterized before and after the proposed treatment using free or fixed bacteria. Characterization of the wastewater included its pH, temperature, dissolved oxygen content (DO), total suspended solids (TSS), total dissolved solids (TDS), biochemical oxygen demand, chemical oxygen demand (COD), and total viable count of bacteria (TVC) all of which were determined using the standard techniques described in Standard Methods for the Examination of Water and Wastewater^14^. After the treatment, residual levels of the selected parameters were determined at each exposure time, and their removal efficiency was calculated to determine the effectiveness of the remediation process according to the following equation:

$${\text{Removal Efficiency }}\left( {{\text{RE }}\% } \right) \, = {\text{ C}}0 \, - {\text{ RC}}/{\text{ C}}0{\text{ X 1}}00$$

where C0 = Initial Concentration before Treatment (Zero Time); RC = Residual Concentration after Treatment at each Exposure Time.

## Results

### Screening batch bioassay using free-living bacteria

**Figure 1:**
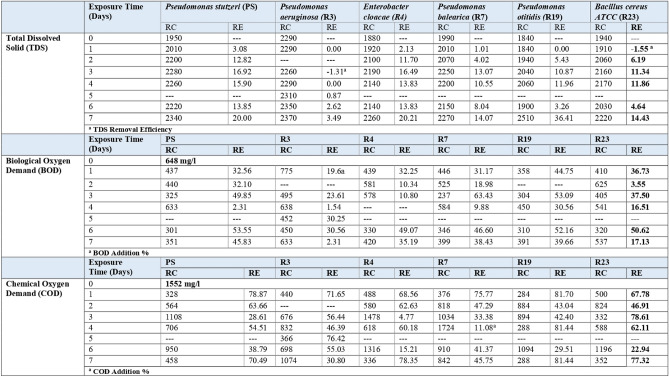
Residual Concentration (RC, mg/l) and removal efficiency (RE %) of Total Dissolved Solid (TDS) in the Treated Oily Wastewater during the Screening Test.

A batch treatment test was performed using the six bacterial species as individuals to select the most promising isolates for the bioremediation process in oily industrial effluents. Samples (50 ml) of the treated effluent were drawn at 24 h intervals, and the quality parameters (temperature, TDS, BOD, COD, Oil and Grease, and TPH) were determined. The following represents residual concentrations (RCs) and removal efficiencies (REs %) of different tested parameters. TDS increased in the treated samples due to the remediation process where biodegradation broke down complex contaminants into simply dissolved salts (Fig. 1). The highest TDS additions by the tested bacteria ranged between a maximum of 36.41% by R19 and a minimum of 3.49% by R3. All were achieved after seven days. BOD residual concentration (RC) and removal efficiency (RE %) in the oily wastewater using the tested bacteria at different exposure times are represented in Fig. 1. The highest BOD REs by the tested bacteria ranged between a maximum of 63.43% by R7 and a minimum of 30.56% by R3 after 3 and 6 days, respectively. COD residual concentration (RC) and removal efficiency (RE %) in the oily wastewater using the tested bacteria at different exposure times are represented in Table 2. Oily industrial wastewater recorded a high COD level of 1552 mg/L. All the tested bacteria achieved relatively higher COD removals than those of the BOD.

### Batch Bioassay Using the Most Promising Bacteria

The two promising bacterial candidates (R4 and R19) previously identified as Enterobacter cloacae 279-56 and Pseudomonas otitis MCC10330 were selected according to the preliminary screening of the six tested bacteria.

### Dissolved Oxygen (DO)

Table 1 illustrates DO residual levels (RL) during oily industrial wastewater batch bioremediation using the selected bacterial cultures at different exposure times. There was a general trend of gradual increasing DO levels with increasing exposure time (till the 7th day) in all cultures, either seeded or not.

**Table 1:** Dissolved Oxygen (DO) Variation during the Batch Treatment Using the Selected Bacteria at Different Exposure Times.

Exposure Time (Days)	Control	R4	R19	Mixed Culture
0	1.5 mg/l			
1	0.76**	1.06	0.93	0.64
2	0.92	0.51	0.44	0.49
3	2.64	0.68	0.47	0.46**
4	5.30	0.35**	0.44**	0.58
6	9.74	2.92	2.80	5.38
7	7.98	5.36	5.19	5.09

Significant values are in Asterisk (**).

### Total Dissolved Solids (TDS)

Remediation using the selected bacteria, either in individuals or in mixed culture, increased TDS levels due to the biodegradation of complex pollutants into simply dissolved salts (Table 2). The exogenous cultures R4, R19, and mixed culture showed a regular increase of TDS levels with increasing exposure time with some fluctuations indicating high activity for breaking down complexes.

**Table 2:** Residual Concentration (RC), Removal Efficiency (RE) and Salt Addition of Total Dissolved Solid (TDS) in the Treated Oily Wastewater during the Batch Treatment.

Time (Days)	Control		R4		R19		Mixed Culture	
	RC	RE (%)	RC	RE (%)	RC	RE (%)	RC	RE (%)
0	1220 mg/l							
1	790	35.25	1800	−47.54	1820	−49.18	1870	−53.28
2	790	35.25	1830	−50.00	1840	−50.82	1890	−54.92
3	790	35.25	1850	−51.64	1840	−50.82	1890	−54.92
4	800	34.43	1880	−54.10^b^	1900	−55.74^b^	1940	−59.02^b^
5	770	36.89	690	43.44^a^	1870	−53.28	1800	−47.54 c
7	740	39.34a	1840	−50.82	1700	−39.34^c^	1900	−55.74

^a^The Highest Removal Efficiency (RE%), ^b^The Highest Salt Addition ( SA%).^c^The Lowest Salt Addition (SA %).

### Total Suspended Solids (TSS)

TSS residual concentration (RC) and removal efficiency (RE %) in the raw and treated oily effluent during the batch treatment using the tested bacteria at different exposure times are presented in Table 3. Oily wastewater recorded low **TSS** level (51 mg/l) at the starting point. No general trends were noticed in the removal of the **TSS** during the batch treatment neither with the time nor among the tested cultures (either exogenous or indigenous). All the cultures achieved extremely high RE (99.9–100%) of **TSS** where control culture achieved complete removal (100%) of the **TSS** followed by **R4, R19** (99.9%) and the mixed culture (99.8%) after 1, 3 and 2 days respectively. According to MPL of the **TSS** (60 mg/l), all of the cultures reached acceptable limit for the effluent safe discharge.

**Table 3:** Residual Concentration (RC), Removal Efficiency (RE) a of Total Suspended Solid (TSS) in the Treated Oily Wastewater during the Batch Treatment.

Time (Days)	Control		R4		R19		Mixed Culture	
	RC	RE (%)	RC	RE (%)	RC	RE (%)	RC	RE (%)
0	51 mg/l							
1	0.01	99.98	0.05	99.9^a^	0.12	99.77	0.13	99.74
2	0.01	99.98	0.08	99.84	0.14	99.73	0.10	99.80^a^
3	0.03	99.94	0.13	99.74	0.05	99.9^a^	0.17	99.67
4	0.07	99.86	0.07	99.86	0.17	99.67	0.17	99.67
5	0.0	100^a^	0.16	99.69	0.15	99.71	0.16	99.69
7	0.03	99.94	0.17	99.67	0.18	99.65	0.15	99.71

^a^The Highest Removal Efficiency (RE%) For Each Culture.

### Oil Content

Oil content biodegradation at different exposure times is presented in Table 4. Raw oily wastewater recorded a high oil content level (727.5 mg/l). R4 and the mixed culture exhibited almost the same highest RE of oil (88.5 and 88.0%, respectively), but the mixed culture showed the advantage of achieving this after only 24 h compared to 3 days by R4. After five days, the culture R19 reached a slightly lower (83.8%) oil RE.

**Table 4:** Residual Concentration (RC), Removal Efficiency (RE) of Biochemical Oxygen Demand (BOD) in the Raw and Treated Oily Wastewater during the Batch Treatment.

Time (Days)	Control		R4		R19		Mixed Culture	
	RC	RE (%)	RC	RE (%)	RC	RE (%)	RC	RE (%)
0	648 mg/l							
1	342.9	47.08^b^	605.7	6.53^b^	612.9	5.42^b^	503.1	22.36^b^
2	143.1	77.92	416.7	35.69	432.0	33.33	383.4	40.83
3	138.6	78.61	358.2	44.72	377.1	41.81	369.0	43.06
4	105.3	83.75	193.5	70.14^a^	274.5	57.64	292.5	54.86
5	91.8	85.83^a^	250.2	61.39	311.4	51.94	171.0	73.61^a^
7	127.8	80.28	231.3	64.31	234.9	63.75^a^	215.1	66.81

^a^The Highest RE%, ^b^The Lowest RE% For Each Culture.

### Biochemical Oxygen Demand (BOD)

BOD residual concentration (RC) and removal efficiency (RE %) exhibited general positive relationship with time by all the tested bacteria as well as control (Table 4). Oily industrial wastewater recorded very high BOD level (648 mg/l) at the starting point. Clear variations were recorded among the tested bacteria for BOD removing. Surprisingly, the highest BOD removal efficiency (85.83%) was achieved by the control culture indicating high biodegradative ability of the indigenous bacteria towards biodegradable organic matter.

### Fat, Oil and Grease (FOG)

Tested bacterial strains exhibited different ability to remove oil and grease from oily wastewater during the batch treatment after 7 exposure days (Table 5). Oily industrial wastewater recorded high oil and grease level of 333 mg/l which is 33.3 fold higher than the MPL (10 mg/l). R19 exhibited the highest FOG removal efficiency (73.27%), followed by R4 (66.82%), R7 (65.77%), R23 (59.16%), PS (56.76%) and finally R3 with the lowest recorded (51.20%) all after 7 days. Although considerable removal of FOG was achieved, the lowest recorded RC of the FOG (89 mg/l) still 8.9 fold higher than FOG MPL.

**Table 5:** Residual concentration (RC) and removal efficiency (RE) of Fat, Oil and Grease (FOG) in the treated oily wastewater during the screening test after seven days.

Strain	RC (mg/l)	RE (%)
Initial Oil Content in the Raw Effluent	333	0.00
*Pseudomonas stutzeri* (PS)	144	56.76
*Pseudomonas aeruginosa* PAO1 (3)	162.5	51.20
*Enterobacter cloacae* 279-56 (4)	110.5	66.82
*Pseudomonas balearica* SP1402 (7)	114	65.77
*Pseudomonas otitidis* MCC10330 (19)	89	73.27
*Bacillus cereus* ATCC 14579 (23)	136	59.16

## Discussion

Among the study, six exogenous strains (PS, R3, R4, R7, R19, and R23) known for their ability to degrade environmental pollutants since where they exhibited high bioremediation capabilities for heavily contaminated soil^12^ were selected based on their excellent biodegradation capabilities towards organic contaminants. Molecular characterization of the two most active exogenous bacteria is *Enterobacter cloacae* 279-56 (4) and *Pseudomonas otitis MCC10330 (R19). Enterobacter cloacae* is a rod-shaped, Gram-negative bacterium belonging to the Enterobacteriaceae family. The size of this bacterium ranges from 0.3–0.6 × 0.8–2.0 μm. *Enterobacter cloacae* live in the mesophilic environment with its optimal temperature at 37 °C and use its peritrichous flagella for movement. This organism is oxidase negative but catalase-positive and is facultatively anaerobic. In other words, this organism can make ATP by aerobic respiration when oxygen is present but can switch to fermentation in the absence of oxygen^15^. *Pseudomonas otitis* is a Gram-negative bacterium that causes otitis^16^. The marvelous resistance and superior potentiality of *Pseudomonas* for biodegradation of toxic organic pollutants and bio-sorption of heavy metals are extensively proved by many authors^17–19^ and many more. The last two strains showed a high ability for remediation of oily- contaminated effluent either as an individual or as a mixture.

Results revealed that a combination of the exogenous microbial strains (bacterial consortium) could enhance the microbial ability, resulting in a high degree of oily wastewater degradation. A reasonable explanation of this phenomenon is that they have synergistic effects and complete each other in the degradation of oil contamination wastewater surfactants where individual strains could not achieve the degradation% obtained by their mixture. It may also be attributed to the production of some bio-surfactant and surface-active compounds (secondary metabolites), which seem to provide nutrients for other bacteria in the consortium, thus enhancing their growth and degradation ability^20^. Oily industrial wastewater used in the present study can be classified as strong since it is highly polluted and contains extremely high levels of the tested contaminants that require powerful treatment to minimize its pollution load and discharge it safely. It was subjected to treatment in a batch experiment using free-living individuals and mixed, time- and species-dependent bacteria. Accordingly, it resulted in varying levels of contaminants removal efficiencies. TDS increased with increasing exposure time with some fluctuations compared to the raw effluent during the present study. These levels are expected to be attributed to the high organic content of the oil wastewater and the bacterial activities of the potent bacteria tested. It is well known that breaking down organic matter increases dissolved salts (TDS) levels. It was found that the removal of organic matter was more affected by changes in salinity than the changes in hydraulic retention time or organic loading rate^21^.

Raw oily wastewater recorded 51 mg/l TSS at the starting point of the experiment. TSS levels resulted from the high organic content in oily effluent. The tested cultures could achieve more than 99.77% (0.12 mg/l) within 24 h, only indicating high degradation ability. This residual concentration is lower than the TSS MPL (60 mg/l). Raw oily industrial wastewater recorded a high BOD level (648.0 mg/l) at the starting point. High RE% of BOD was recorded among all the tested bacteria, with clear variations. The control with its indigenous bacteria recorded the highest RE (85.83%) after (5) exposure days with 91.8 mg/l RC. For exogenous bacteria, the highest RE (73.61%) was recorded by the *mixed culture* after (5) exposure days with 171 mg/l RC that is exceeding the MPL (60 mg/l). Raw effluent also contained a high COD level (1552 mg/l). Very high COD removal was achieved where, again, the control culture showed the highest RE (99.03%) after four exposure days, reaching 15 mg/l. The highest RE recorded was 91.56% among exogenous bacteria by the *mixed culture*. This is equivalent to 131 mg/l, which is close to the MPL (100 mg/l). This high removal by the control culture for COD and BOD indicates the high degradative ability of the indigenous bacteria towards the chemically degraded and biodegradable organic matter. Some of these results did not reach the safe discharge limit according to MPL. It could be due to the operation mode (batch) with the short exposure, which is also confirmed by other workers using the continuous operation mode for treating effluents rich in organic load. It is always expected that continuous treatment enhances the RE of the included contaminants compared to batch operation. These facts are supported by other workers who reported a 75% increase in the RE of COD with increasing sludge retention time (SRT) to 15 days^22^. Other workers reported 60 to 90% of total nitrogen, 90% in COD, and 100% in ammonium removal using a membrane reactor^23^. No pre- or post-treatment were used in the present study, giving the proposed treatment another advantage. The raw Oil-contaminated effluent tested in the batch experiment recorded a very high Oil Content level (727.5 mg/l). All the tested species achieved remarkably high REs of Oil Content except the unseeded wastewater. The highest Oil Content removal (88.5%) was recorded by R4, equivalent to (84 mg/l).

This result is very promising considering the treatment duration, efficiency, cost, operation conditions, energy used, and impact on the environment compared to other chemical treatments where cost is increased due to the chemicals used, energy input, or removal of sludge produced. A similar study by Al-Malack^23^ aimed to minimize the Oil Content level, where an immersed membrane process was used to remove Oil Content from highly pollutant petroleum refinery wastewater, and the maximum removal efficiency recorded was 69%. In another study by Asatekin and Mayes^24^, a physiochemical treatment was applied using ultra-filtration (UF) membranes to treat refinery wastewater where the highest recorded removal efficiency of COD was 44%. In addition, poly aluminum chloride and ferric chloride for coagulation treatment of petroleum wastewater reached 58% removal of COD^25^. Furthermore, ferric chloride (FeCl3) could achieve 52% COD removal. Moreover, in subsequent chemical coagulation by hydrogen peroxide, 58 and 78% removal efficiencies for COD and BOD, respectively, were achieved, Wagner and Nicell^26^. In the present study, 85.83, 99.03, and 88.5% REs of BOD, COD, and oil Content, respectively, were achieved by using biological treatment only with no chemical treatments were used, confirming the superiority of the proposed treatment efficiency, especially at the very high Oil Content and COD concentrations in the raw effluents (727.5 and 1552 mg/l respectively). Present study lack TOC analysis which is the main limitation of present study.

## Conclusion

Oily industrial wastewater showed extremely high levels of all the tested pollutants, especially organic and inorganic, making it one of the strongest industrial effluents with many difficulties in its treatment and dangerous effects on the receiving environments.

## Data Availability

The data set generated from this study will be available on a reasonable request from the corresponding author.

## References

[1] Hossain L, Sarker SK, Khan MS (2018). Evaluation of present and future wastewater impacts of textile dyeing industries in Bangladesh. Environ. Dev..

[2] Marcus AC, Ekpete OA (2014). Impact of discharged process wastewater from an oil refinery on the physicochemical quality of a receiving water body in rivers state Nigeria. J. Appl. Chem.

[3] Wu M (2017). Bioremediation of hydrocarbon degradation in a petroleum-contaminated soil and microbial population and activity determination. Chemosphere.

[4] Marchand C, St-Arnaud M, Hogland W, Bell TH, Hijri M (2017). Petroleum biodegradation capacity of bacteria and fungi isolated from petroleum-contaminated soil. Int. Biodeterior. Biodegrad..

[5] Nagda A, Meena M, Shah MP (2022). Bioremediation of industrial effluents: A synergistic approach. J. Basic Microbiol..

[6] Jain PK, Bajpai V (2012). Biotechnology of bioremediation-a review. Int. J. Environ. Sci..

[7] Hesnawi, R, K. Dahmani, K, Al-Swayah, A, S. Mohamed, S, Mohammed, S.A. Biodegradation of municipal wastewater with local and commercial bacteria, 12th International Conference on Computing and Control for the Water Industry, CCWI2013, Procedia Engineering, **70**: 810–814 (2014).

[8] Kushwaha JP, Srivastava VC, Mall ID (2011). An overview of various technologies for the treatment of dairy wastewaters. Crit. Rev. Food Sci..

[9] Kelly PT, Zhen H (2014). Understanding the application niche of microbial fuel cells in a cheese wastewater treatment process. Bioresour. Technol..

[10] Deegan AM, Shaik B, Nolan K, Urell K, Oelgemoller M, Tobin J, Morrissey A (2011). Treatment options for wastewater effluents from pharmaceutical companies. Int. J. Environ. Sci. Technol..

[11] Vanerkar AP, Satyanarayan S, Dharmadhikari DM (2013). Full scale treatment of herbal pharmaceutical industry wastewater. Int. J. Chem. Phys. Sci..

[12] El-Bestawy E, Sabir J, Mansy A (2013). H & Zabermawi, nisolation, identification, and acclimatization of atrazine-resistant soil bacteria. Ann. Agri. Sci..

[13] El Bestawy E, Abu Rass M, Abdel-Kawi MA (2013). Removal of lead and oil hydrocarbon from oil refining-contaminated wastewater using Pseudomonas spp. J. Nat. Sci. Res..

[14] Clesceri, L.S., Greenberg, C.G. and Eaton, A.D. Standard Methods for the Examination of Water and Wastewater, 20th edn. USA. In *American Public Health Association (APHA).* ISBN 0875532357 (1999).

[15] Berney M, Greening C, Conrad R, Jacobs WR, Cook GM (2014). An obligately aerobic soil bacterium activates fermentative hydrogen production to survive reductive stress during hypoxia. Proc. Nat. Acad. Sci..

[16] Roland PS, Stroman DW (2002). Microbiology of acute otitis externa. Laryngoscope.

[17] Mohammed, A. S, Kapri, A & Goel, R. Heavy metal pollution: source, impact, and remedies. In *Bio management of metal-contaminated soils* (1–28). Springer*,* Dordrecht. (2011).

[18] Gupta VK, Nayak A, Agarwal S (2015). Bioadsorbents for remediation of heavy metals: Current status and their prospects. Environ. Eng. Res..

[19] Kanamarlapudi SLRK, Chintalpudi VK, Muddada S (2018). Application of biosorption for the removal of heavy metals from wastewater. Biosorption.

[20] Banat IM, Carboué Q, Saucedo-Castañeda G, de JesúsCázares-Marinero J (2021). Biosurfactants: The green generation of specialty chemicals and potential production using solid-state fermentation (SSF) technology. Bioresour. Technol..

[21] Lay WC, Liu Y, Fane AG (2010). Impacts of salinity on the performance of high retention membrane bioreactors for water reclamation: A review. Water Res..

[22] Bolzonella D, Pavan P, Battistoni P, Cecchi F (2005). Mesophilic anaerobic digestion of waste activated sludge: Influence of the solids retention time in the wastewater treatment. Process Biochem..

[23] Al-Malack MH, Basaleh AA (2016). Adsorption of heavy metals using activated carbon produced from municipal organic solid waste. Desalin. Water Treat..

[24] Asatekin A, Mayes AM (2009). Oil industry wastewater treatment with fouling resistant membranes containing amphiphilic comb copolymers. Environ. Sci. Technol..

[25] Farajnezhad H, Gharbani P (2012). Coagulation treatment of wastewater in the petroleum industry using poly aluminum chloride and ferric chloride. Int. J. Res. Rev. App Sci..

[26] Wagner M, Nicell JA (2001). Peroxidase-catalyzed removal of phenols from a petroleum refinery. Water Sci. Technol..

